# Analysis of the surprise question as a tool for predicting death in neonates

**DOI:** 10.1007/s00431-024-05879-8

**Published:** 2025-02-07

**Authors:** Silvia Asenjo, Aleix Soler-Garcia, Ana Morillo-Palomo, Anna Habimana-Jordana, Montserrat Guillen, Catalina Bolancé, Sergi Navarro-Vilarrubí

**Affiliations:** 1 Department of Pediatric Palliative Care, Hospital Sant Joan de Déu, Barcelona, Spain; 2 Pediatrics Department, Hospital Sant Joan de Déu, Passeig de Sant Joan de Déu 2, 08950 Esplugues de Llobregat, Barcelona, Spain; 3 Neonatology Department, Sant Joan de Déu Hospital, Barcelona, Spain; 4 Department of Econometrics, Statistics and Applied Economics, University of Barcelona, Barcelona, Spain; 5 Infectious Diseases and Microbiome Research Group, Institut de Recerca Sant Joan de Déu, Esplugues de Llobregat, Spain; 6 Departament de Cirurgia i Especialitats Medicoquirúrgiques, Facultat de Medicina i Ciències de la Salut, Universitat de Barcelona, Barcelona, Spain

**Keywords:** Palliative care, Surprise Question, Neonatology, Predictor factor

## Abstract

**Abstract:**

The Surprise Question “Would you be surprised if the patient died in the next 12 months?” lacks pediatric research, particularly in neonatal patients. Our study aims to analyze the Surprise Question’s predictive ability in neonates and explore pediatricians’ views on palliative care patient identification. A prospective cross-sectional study was conducted from February 2021 to June 2023, including all newborns admitted to the Neonatal Intensive Care Unit of a pediatric tertiary hospital and its pediatricians. Patients with less than a year since admission were excluded from final analyses. Recorded variables included patient demographics and condition, pediatricians’ profile and opinions regarding the Surprise Question, and palliative care patient identification. The Surprise Question was formulated to one or more pediatricians per neonate at admission, 7 days of life, and 28 days of life, with patient status recorded after 12 months to elaborate a confusion matrix of prognostic test results. A total of 51 pediatricians participated. Most felt they had limited criteria for identifying palliative care patients (55%), believed the Surprise Question could be useful (77%), and predicted death (75%). The Surprise Question was answered at least in one out of the three moments for 262 neonates (61% male and at least 36% preterm), with sufficient sample at each moment to study its predictive ability. High negative predictive values were consistent, with higher positive predictive value at 7 days (26%).

**Conclusions:**

The Surprise Question is a promising tool for predicting neonatal outcome and could guide professionals in initiating palliative care discussions. The 7-day mark appears more suitable for this application.

**Table:** 

**What is Known:** *• Previous research has established the Surprise Question as a valuable tool for predicting death in adults. However, limited research exists on its use in pediatric patients and its role remains unexplored in the neonatal period.*	
**What is New:** *• The study evaluates the Surprise Question as a tool for predicting death within the first year of life when applied in the neonatal period. It offers insights into its predictive ability and most suitable time for its application. This study sheds light on its applicability in neonatal care, offering a valuable tool for early identification and referral to palliative care.*

**Supplementary information:**

The online version contains supplementary material available at 10.1007/s00431-024-05879-8.

## Introduction

The Surprise Question (SQ) is a tool used in palliative care or in certain situations of uncertainty in which there is a real possibility of death in a patient. This tool is based on the answer from the patient’s physician to the question, “Would it surprise you if the patient were to die in the next 12 months?” Thus, the SQ evaluates how surprising it would be if an adverse event occurred, instead of focusing on how much more likely would it be. Therefore, it is a tool created to provide sensitivity, to the detriment of less specificity. Indeed, in adults, the SQ has been used as a predictor of the risk of death to identify the need for a referral to palliative care services. In relation to this, recent analyses present disparate results, with sensitivity ranging from 67 to 81% and specificity from 51 to 81%. However, they agree in presenting high negative predictive values (proportion of patients who survive when the SQ is unexpected, meaning death is not expected), with lower positive predictive values (deaths when the SQ is expected, meaning death is expected) [1–6]. Some studies describe greater accuracy in the context of oncological patients compared to other types of diseases [1], and they generally agree on the need for further studies and the possibility of using the SQ in combination with other scales or tests to improve its utility [2–6]. There are few studies that analyze the SQ in pediatrics, highlighting those developed by Burke et al. [7] in patients aged between 0 and 20 years and by Alizadeh et al. [8] in patients aged between 1 month and 19 years with advanced heart disease. However, to our knowledge, there are no studies where the SQ is exclusively applied in the neonatal period. Therefore, the objective of our study is to analyze to what extent the SQ can predict death during the first year of life when posed in the neonatal period.

## Methods

This is a prospective cross-sectional study conducted between February 2021 and June 2023 in the Neonatal Intensive Care Unit (NICU) of a tertiary-level pediatric hospital. Data were collected through the Research Electronic Data Capture (Redcap) platform. The study was approved by the Hospital Sant Joan de Déu Ethics Committee (Esplugues, Spain; refPIC-177–20) and was done in accordance with the Declaration of Helsinki. Families signed a generic consent for the use of data for research upon admission to the NICU. All neonates admitted to the NICU from the beginning of the study until June 2022 were unselectively and prospectively included, and data analysis was delayed by 12 months to assess the patients’ status (alive/*exitus*) at 1 year of age. For each patient, neonatologists from the unit (distinguishing between those who were the patient’s primary caregivers and those who were not) were asked to answer the SQ at three moments in time: upon admission to the NICU, at 7 days of life, and at 28 days of life (if still admitted to the NICU). Data regarding patient sex and main pathology were also collected. Lastly, the following variables of the answering neonatologists were collected: sex, age, professional category and experience, and self-perceived ability to detect the indication to initiate palliative care (PC). The questions were answered anonymously, so each professional was identified by a personal code known only to themselves, which they maintained throughout the study. In cases of disagreement between physicians regarding the response to the SQ for a given patient, the majority’s response was established for each patient and time of response. In case of a tie, the response of the neonatologist who indicated belonging to the team responsible for the patient was selected. Information regarding the patients’ status (alive/*exitus*) at 1 year of age was extracted from reviewing their medical records. Responses to categorical variables were presented as proportions along with their 95% confidence interval (CI), calculated using Agresti and Coull [9]. The results to the question “Would you be surprised if the patient died in the next year?” were classified into expected death (SQ expected, when the response is “no”) and unexpected death (SQ unexpected, when the response is “yes”). The sensitivity of the SQ was calculated for each time moment based on the proportion of patients with expected death at SQ at that time among those who died within the first year of life. Similarly, the specificity of the SQ was quantified for each time point by calculating the proportion of patients with a non-expected death at SQ among those who survived after 1 year. The positive predictive value (PPV) was defined as the proportion of deaths among patients with an expected SQ, while the negative predictive value (NPV) was defined as the ratio of surviving patients among those with an unexpected SQ. Accuracy was calculated as the total proportion of patients (alive or *exitus*) correctly predicted by the SQ. Hypothesis testing was performed and *p*-values below 0.05 were considered significant. Statistical analysis was performed using R version 4.3.2 for Windows (R Core Team. R: A language and environment for statistical computing. R Foundation for Statistical Computing, Vienna, Austria. 2024. URL https://www.R-project.org/).

## Results

Information was available at least at one time point (upon admission, 7 days of life, or 28 days of life) for a total of 262 neonates, 61.1% of whom were male. The main pathologies were prematurity (36%), congenital heart disease (21%), and pulmonary illness (20%). The number of responses obtained for the SQ was 784 (3.0 responses per patient on average). Consensus in responses was achieved for 226 (86.3%) of the patients. Table 1 shows the main characteristics overall and at each of the time point when the SQ was performed.

**Table 1:** Absolute frequencies and percentages of neonates at admission, at 7 days, and at 28 days, by sex and pathology

	Total patients		Admission		7 days		28 days	
**Total**	*N*	Percentage	*N*	Percentage	*N*	Percentage	*N*	Percentage
	262	100.0%	201	100.0%	138	100.0%	56	100.0%
**Sex**								
Male	160	61.1%	133	66.2%	80	58.0%	28	50.0%
Female	102	38.9%	68	33.8%	58	42.0%	28	50.0%
**Pathology**								
Nervous system	24	9.2%	23	11.4%	8	5.8%	0	0.0%
Pulmonary	52	19.9%	41	20.4%	23	16.7%	4	7.1%
Heart disease	55	21.0%	42	20.9%	39	28.3%	16	28.6%
Prematurity	93	35.5%	64	31.8%	50	36.2%	35	62.5%
Others	38^a^	14.5%	31	15.4%	18	13.0%	1	1.8%

^a^The main reasons for admission were sepsis (*n* = 5) and hyperbilirubinemia (*n* = 5)

**Fig. 1:**
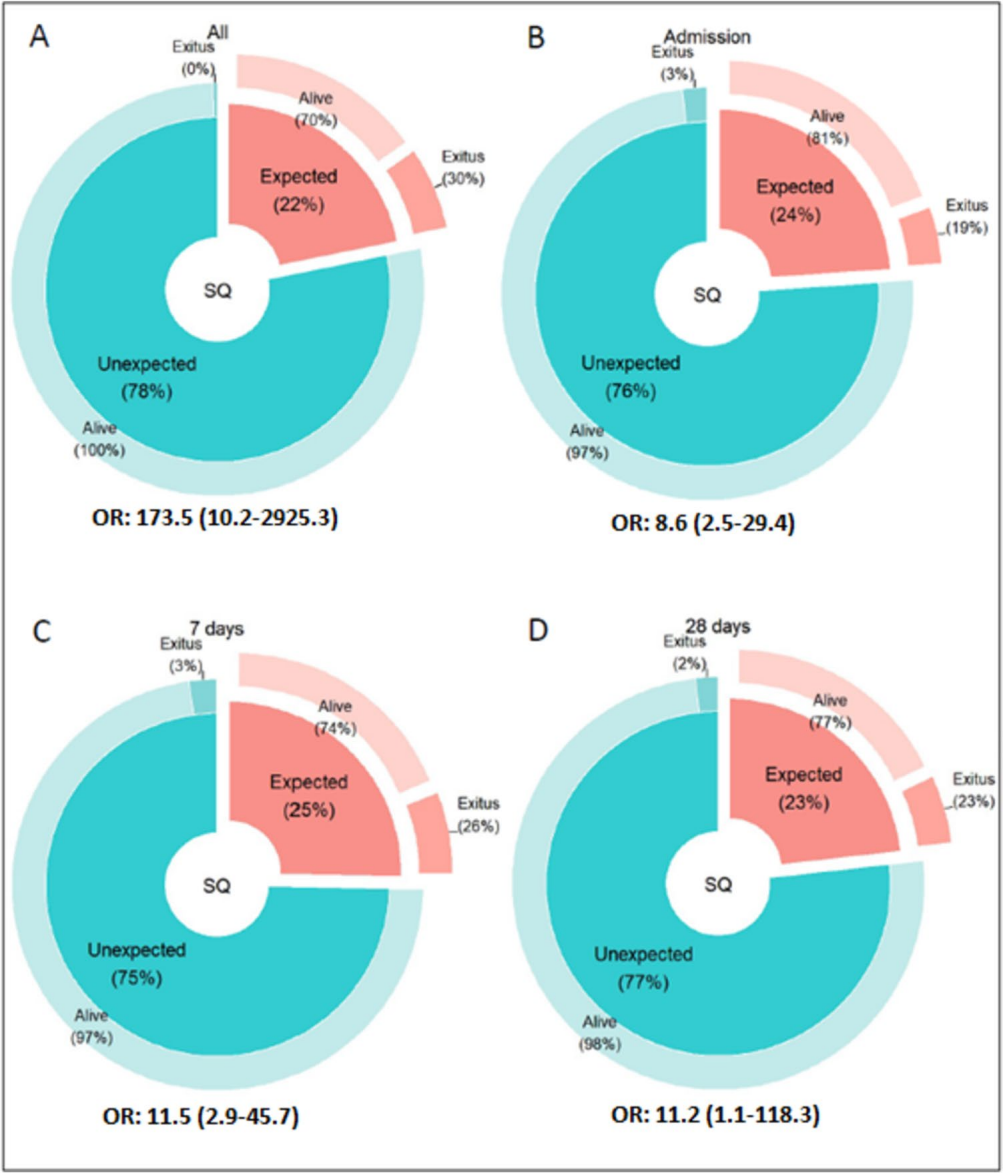
Representation of patients’ status and odds ratio of exitus at 1 year of age according to the response to the SQ at different moments in time. Footnote: *Graph **A** represents the global result for the SQ. For the global result, the patient classifies as SQ expected if that is the answer for at least one moment in time. Graphs **B**, **C**, and **D** represent results at admission, 7 days of life, and 28 days of life, respectively. For the odds ratio calculation, when zeros caused problems, 0.5 was added to all cells, according to previous statistical models.^10^ OR, odds ratio

Among the patient cohort, a total of 18 (6.9%) had deceased within 12 months of their birth. On the other hand, among the 262 patients, 57 (21.8%) had an expected SQ at some point, among whom 17 (29.8%) died, meaning approximately one in three who had an expected SQ at some point died before 12 months. Lastly, among the 205 (78.2%) who had an unexpected SQ at all time points, 1 (0.5%) had died before a year. Figure 1 shows the results of the SQ and survival globally and for each one of the evaluated moments in time. Neither the patient’s status at 12 months (alive/*exitus*) nor the SQ outcome varied significantly based on the timing of the SQ (*p* = 0.740 for status and *p* = 0.368 for SQ outcome). Regarding its diagnostic performance, the SQ showed sensitivity, specificity, PPV, and NPV of 69.2–75.0%, 78.9–79.4%, 18.8–25.7%, and 97.1–97.6%, respectively, varying depending on the timing of the SQ response. Table 2 details these results. As observed, the highest diagnostic performance was obtained at 7 days of life. There were no significant differences in results between premature patients and those with other types of pathologies (see Table S1) for any of the parameters evaluated and for any response time.

**Table 2:** Confusion matrix of prognostic test results (95% CI)

	Sensitivity	Specificity	PPV	NPV	Accuracy	*N*^a^
Admission	69.2 (38.6–90.9)	79.3 (72.8–84.8)	18.8 (9.0–32.6)	97.4 (93.4–99.3)	78.6 (72.3–84.1)	201
7 days	75.0 (42.8–94.5)	79.4 (71.3–86.1)	25.7 (12.5–43.3)	97.1 (91.7–99.4)	79.0 (71.2–85.5)	138
28 days	75.0 (19.4–0.99.4)	78.9 (65.3–88.9)	21.4 (4.7–50.8)	97.6 (87.4–99.9)	78.6 (65.6–88.4)	56

^a^Number of patients with SQ at admission, 7 days of life, and 28 days of life

The characteristics of the 51 neonatologists who participated in the study, majority of whom were female (78.3%) and less than 40 years of age (72.3%), are presented in Table S2. Responses to questions regarding PC and the SQ are also documented.

## Discussion

The sample obtained in our study resembles that of other relevant studies in this field, such as those conducted by Burke et al. (2018)^7^ and Alizadeh et al. (2022)^8^, with results of similar magnitude to those described by them in the pediatric population. This study contributes to the existing information on the applicability of the SQ in the pediatric age group, with encouraging results. Moreover, at the time of its writing, there are no other published studies exclusively conducted in the neonatal population. Given the complexity, pluripathology, and vulnerability of the patient profile in a NICU, early identification of patients eligible for PC is essential to ensure adequate care and to provide comprehensive care for patients and their families. As previously mentioned, the way the SQ is formulated aims to highlight its sensitivity. Indeed, we observed high levels of sensitivity, which means that SQ performs well as a tool for the early detection of these patients. This can fulfill the professionals’ need for a detection tool since in the initial survey, 55% considered having little criteria for it. Our results also suggest that 7 days of life might be the best time to apply the SQ as it has the highest PPV (25.7%, 95% CI 12.5–43.3), meaning one death for every four expected SQ at 7 days of life. However, according to the obtained 95% CI, the other time points have similar PPV, meaning that each time point might be equally appropriate.

The study findings suggest that SQ may become a routine assessment tool in neonatal and pediatric care, helping to guide healthcare professionals in initiating discussions about palliative care needs and end-of-life planning. The role of intensive care pediatricians and neonatologists is crucial for early initiation of comprehensive PC and interdisciplinary teamwork to address the needs of patients and families.

Regarding the limitations of the study, firstly, it is worth mentioning that this is a single-center study conducted at a tertiary and single-specialty pediatric hospital. There could also be a response bias, as it is possible that professionals responded more frequently in cases of greater complexity and severity. We do not have information on the actual fact that some professionals may not have answered the SQ at all the considered time points. Likewise, the availability of responses at the three time points was variable. However, we have a sufficient sample at each time point to analyze the predictive capacity of the tool we evaluated.

In conclusion, our study provides significant insights into the application of the SQ as a tool for detecting neonates eligible for palliative care. Despite some limitations, our results support the usefulness of the detection tool and suggest that performing the SQ in the seventh day of life shows a good performance. The study opens the door to future research on the use of the SQ in newborns and pediatric patients. Future studies could explore the applicability of the SQ in different clinical contexts and define the timing of application to achieve optimal predictive accuracy.

## Supplementary information

Below is the link to the electronic supplementary material.

Supplementary file1 (DOCX 14 KB)

## Data Availability

No datasets were generated or analysed during the current study.

## Abbreviations

CI: Confidence interval
NICU: Neonatal Intensive Care Unit
NPV: Negative predictive value
PC: Palliative care
PPV: Positive predictive value
SQ: Surprise Question
