# Supplementary material for: Analysis of the surprise question as a tool for predicting death in neonates
Source: Eur J Pediatr. 2025 Feb 7;184(2):182. doi: 10.1007/s00431-024-05879-8 (PMC11805820; doi:10.1007/s00431-024-05879-8)
Supplement: Supplementary file 1 — Supplementary file1 (DOCX 14 KB) [file 431_2024_5879_MOESM1_ESM.docx]

**Table S1:** Confusion Matrix comparing premature patients with those admitted for another reason. The comparison is performed using chi-square test or Fisher's exact test depending on the sample sizeTop of Form

**Bottom of Form**

|  | | Sensitivity | | Specificity | | PPV | | NPV | | Accuracy | | n | |
| --- | --- | --- | --- | --- | --- | --- | --- | --- | --- | --- | --- | --- | --- |
|  |  | Value | p-value | Value | p-value | Value | p-value | Value | p-value | Value | p-value |  |  |
| Admission | Non-premature | 63,6  (30,8-89,1) | 1,000 | 82,5  (74,8-88,7) | 0,113 | 24,1  (10,3-43,5) | 0,286 | 96,3  (90,8-99,0) | 0,321 | 81,0  (73,4-87,2) | 0,222 | 137 | 201 |
|  | Premature | 100  (15,8-100) |  | 72,6  (59,8-83,2) |  | 10,5  (1,3-33,1) |  | 100  (92,1-100) |  | 73,4  (60,9-83,7) |  | 64 |  |
| 7 days | Non-premature | 75,0  (42,8-94,5) | No calculable | 75,0  (63,7-84,2) | 0,178 | 32,1  (15,9-52,3) | 0,153 | 95,0  (86,1-99,0) | 0,263 | 75,0  (64,6-83,6) | 0,127 | 88 | 138 |
|  | Premature | NP^1^ |  | 86,0  (73,3-94,2) |  | 0,0  (0,0-41,0) |  | 100  (91,8-100) |  | 86,0  (73,3-94,2) |  | 50 |  |
| 28 days | Non-premature | 100  (29,2-100) | 0,250 | 66,7  (41,0-86,7) | 0,190 | 33,3  (7,5-70,1) | 0,258 | 100  (73,5-100) | 1,000 | 71,4  (47,8-88,7) | 0,334 | 21 | 56 |
|  | Premature | 0,0  (0,0-97,5) |  | 85,3  (68,9-95,1) |  | 0,0  (0,0-52,2) |  | 96,7  (90,2-100) |  | 82,9  (66,3-93,4) |  | 35 |  |

^1^ No deaths in the group, therefore sensitivity cannot be calculated, making the p-value incalculable
